# Primary Human Derived Blood Outgrowth Endothelial Cells: An Appropriate In Vitro Model to Study Shiga Toxin Mediated Damage of Endothelial Cells

**DOI:** 10.3390/toxins12080483

**Published:** 2020-07-29

**Authors:** Wouter J. C. Feitz, Nicole C. A. J. van de Kar, Ian Cheong, Thea J. A. M. van der Velden, Carolina G. Ortiz-Sandoval, Dorothea Orth-Höller, Lambert P. J. W. van den Heuvel, Christoph Licht

**Affiliations:** 1 Department of Pediatric Nephrology, Amalia Children’s Hospital, Radboud Institute for Molecular Life Sciences, Radboudumc, 6525 GA Nijmegen, The Netherlands; Wouter.Feitz@radboudumc.nl (W.J.C.F.); nicole.vandeKar@radboudumc.nl (N.C.A.J.v.d.K.); thea.vandervelden@radboudumc.nl (T.J.A.M.v.d.V.); bert.vandenheuvel@radboudumc.nl (L.P.J.W.v.d.H.); 2 Cell Biology Program, Research Institute, The Hospital for Sick Children, Toronto, ON M5G 1X8, Canada; icheong2@uwo.ca (I.C.); carolina.ortiz@sickkids.ca (C.G.O.-S.); 3 Division of Hygiene and Medical Microbiology, Medical University of Innsbruck, 6020 Innsbruck, Austria; dorothea.orth@i-med.ac.at; 4 Department of Development and Regeneration, Department of Pediatric Nephrology, KU, 3000 Leuven, Belgium; 5 Division of Nephrology, The Hospital for Sick Children, Toronto, ON M5G 1X8, Canada; 6 Department of Pediatrics, University of Toronto, Toronto, ON M5G 1X8, Canada

**Keywords:** hemolytic uremic syndrome, shiga toxin, blood outgrowth endothelial cells

## Abstract

Hemolytic uremic syndrome (HUS) is a rare disease primarily characterized by hemolytic anemia, thrombocytopenia, and acute renal failure. Endothelial damage is the hallmark of the pathogenesis of HUS with an infection with the Shiga toxin (Stx) producing *Escherichia coli* (STEC-HUS) as the main underlying cause in childhood. In this study, blood outgrowth endothelial cells (BOECs) were isolated from healthy donors serving as controls and patients recovered from STEC-HUS. We hypothesized that Stx is more cytotoxic for STEC-HUS BOECs compared to healthy donor control BOECs explained via a higher amount of Stx bound to the cell surface. Binding of Shiga toxin-2a (Stx2a) was investigated and the effect on cytotoxicity, protein synthesis, wound healing, and cell proliferation was studied in static conditions. Results show that BOECs are highly susceptible for Stx2a. Stx2a is able to bind to the cell surface of BOECs with cytotoxicity in a dose-dependent manner as a result. Pre-treatment with tumor necrosis factor alpha (TNF-α) results in enhanced Stx binding with 20–30% increased lactate dehydrogenase (LDH) release. Endothelial wound healing is delayed in a Stx2a-rich environment; however, this is not caused by an effect on the proliferation rate of BOECs. No significant differences were found between control BOECs and BOECs from recovered STEC-HUS patients in terms of Stx2a binding and inhibition of protein synthesis.

## 1. Introduction

The endothelium is the largest organ in our body consisting of endothelial cells lining the inner surface of blood vessels [1]. The vascular endothelium is actively involved in dynamic repair and regulatory processes ultimately aiming to maintain vascular homeostasis. Endothelial cells not only function to separate the vascular lumen from the surrounding tissue, but also function as regulators of permeability and smooth muscle tone, control immune and inflammatory responses, and play important roles in angiogenesis, hemostasis, and coagulation [1,2].

Hemolytic uremic syndrome (HUS) is a rare disease primarily characterized by hemolytic anemia, thrombocytopenia, and acute renal failure [3,4]. Endothelial damage in the glomerular capillaries of the kidney plays a central role in the pathogenesis of HUS [2]. HUS is typically caused by an infection with the Shiga toxin (Stx) producing *Escherichia coli* (STEC-HUS), and represents one of the major causes of acute renal failure in childhood [4]. The bacteria are typically hosted in cattle and infection occurs by ingestion of contaminated food products, but also via direct person-to-person or animal contact [5]. After ingestion, the Stx producing *E. coli* colonize the gut, damage the intestinal epithelium and cause bloody diarrhea in 80–90% of cases [6]. Stx may subsequently enter the bloodstream, which allows the bacterial toxin to bind to its main receptor, the globotriaosylceramide (Gb3) receptor, ubiquitously present on endothelial cells (e.g., the glomerular capillaries) [7,8,9,10]. The toxin is internalized via endocytosis with protein inhibition and apoptosis of cells as a result [6]. This leads to endothelial cell death, followed by the formation of microthrombi, occlusion of the glomerular microvasculature, and ultimately renal failure. While there is no specific treatment for STEC-HUS, about 70% of patients recover spontaneously upon supportive therapy alone [11]. However, around 25% of patients have long-term sequelae with proteinuria, microalbuminuria, and hypertension and permanent end stage renal disease (ESRD) occurs in 12% of cases [12,13]. The mortality rate in the acute phase of disease is 2–5%. Typically, STEC-HUS is a disease with no recurrence risk [13,14].

In the past, endothelial cell lines, like human umbilical vein endothelial cells (HUVECs) and glomerular microvascular endothelial cells (GMVECs), have been used in experimental studies to investigate the pathogenesis of HUS [9,10]. However, the interaction of Stx with blood outgrowth endothelial cells (BOECs) has not yet been studied. BOECs are (likely) marrow-derived endothelial progenitor cells [15]. These cells can be isolated and cultured from the peripheral blood of a donor and provide the unique advantage to study the endothelial characteristics of individuals with different genetic backgrounds. They have a typical cobblestone morphology, are highly proliferative, can be expanded over multiple passages with retention of their endothelial cell characteristics, and promote de novo vessel formation in vivo [16,17]. It is known that an estimated 15% of children infected with Stx develop HUS [7]. However, the reason why certain children develop this disease and others do not is still unknown. As BOECs harbor the genetic signature of the donor, it makes these cells a highly interesting tool to study the role of the host endothelium in the pathophysiology of STEC-HUS by using BOECs derived from patients with STEC-HUS.

In this study, BOECs from healthy controls and three patients recovered from acute STEC-HUS were isolated. We tested the hypothesis that BOECs derived from patients with STEC-HUS are more sensitive to Stx. In particular, we investigated if such susceptibility was possibly explained by a higher amount of Stx bound to the cell surface. Cells were characterized for endothelial origin, the binding and cytotoxicity of Stx was studied, and the functional consequences of Stx incubation for the endothelium were investigated.

## 2. Results

### 2.1. BOECs Maintain an Endothelial Cell Phenotype

**Figure 1:**
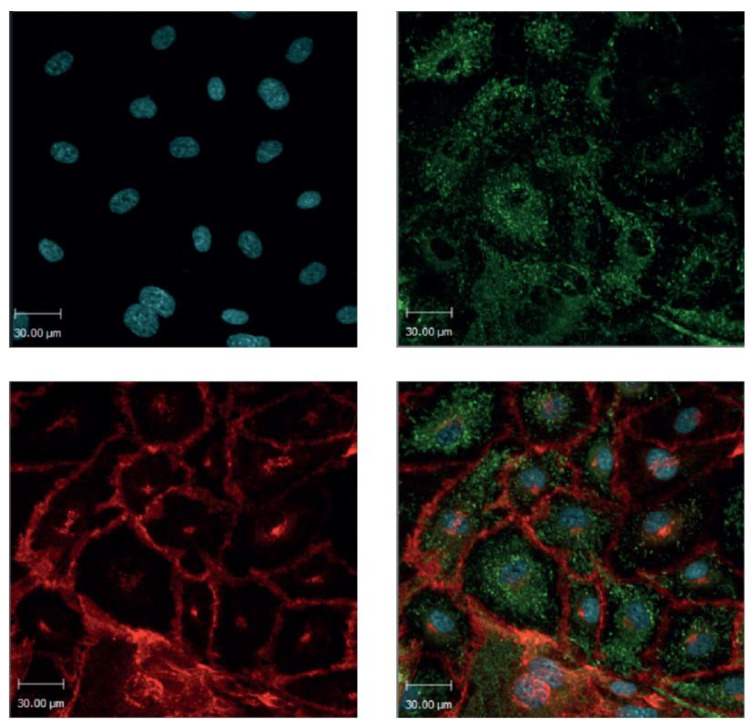
Blood outgrowth endothelial cells. Healthy donor blood outgrowth endothelial cells (BOECs) are stained for the specific endothelial markers Von Willebrand factor (VWF; green) and vascular endothelial cadherin (VECAD; red). The nucleus is stained with the fluorescent DNA stain Hoechst 33342 (blue). The isolated BOECs maintain an endothelial cell phenotype. 20× magnification.

Isolated human BOECs showed a cobblestone morphology and high proliferation rate typical for endothelial cells [17]. Healthy control BOECs stained positively for the specific endothelial cell markers Von Willebrand factor (VWF) and vascular endothelial cadherin (VECAD/CD144) (Figure 1). In conclusion, the obtained BOECs expressed an endothelial cell phenotype.

### 2.2. Shiga Toxin Binding and Cytotoxicity

**Figure 2:**
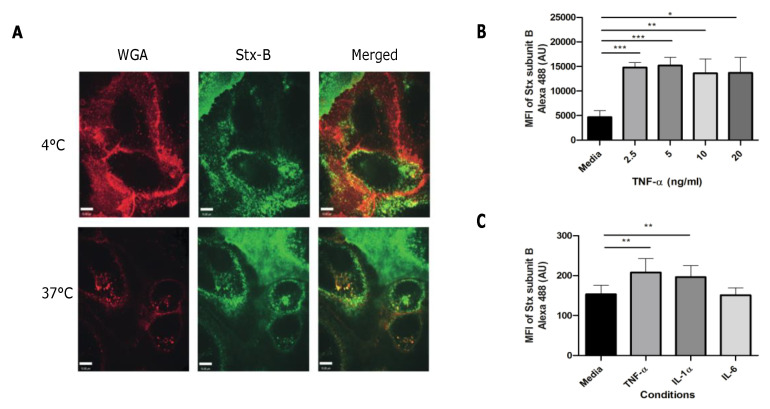
Binding of Shiga toxin subunit B on BOECs. (**A**) Confocal live images of healthy donor BOECs incubated with Alexa 647 labeled WGA (red) to stain the glycocalyx and Alexa 488 labeled Stx-B (green) to study the binding of Stx-B on the cell surface of BOECs (N = 1). (**B**,**C**) BOECs were incubated with different concentrations of TNF-α, 1 ng/mL of IL-1α or 1 ng/mL of IL-6 for 24 h (N = 3 for 2B and N = 7 for 2C). Binding was measured with the use of Alexa 488 labeled Stx-B in combination with flow cytometry. Stx-B is located on the cell surface of BOECs at 4 °C. Stx-B gets internalized by endocytosis at 37 °C. Increased binding of Stx-B was measured when cells were pre-stimulated with TNF-α or IL-1α. Mean values and SEM are given. *p*-Values of <0.0001 (***), <0.001 (**), and <0.05 (*) by *t*-test are indicated. MFI = mean fluorescence intensity; WGA = wheat germ agglutinin; Stx-B = Shiga toxin subunit B; TNF-α = tumor necrosis factor alpha; IL = interleukin.

**Figure 3:**
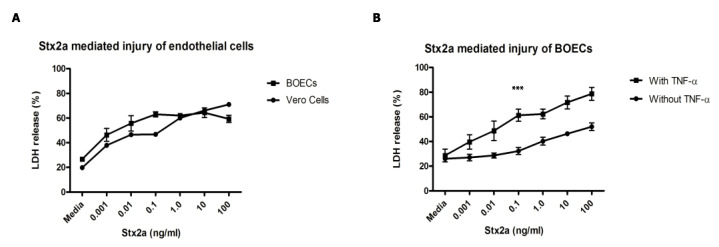
The cytotoxic effect of Stx2a on BOECs. (**A**) Cells were incubated with increasing concentrations of Stx2a for 48 h. Vero cells were used as control cell line (N = 3). (**B**) BOECs were pre-incubated with or without 10 ng/mL of TNF-α for 24 h and incubated with different concentrations of Stx2a for 24 h (N = 3). The release of LDH was measured with a LDH cytotoxicity assay kit. Stx2a is cytotoxic for BOECs in a concentration-dependent manner and pre-incubation with TNF-α increased this effect significantly. A *p*-value of < 0.0001 by ANCOVA is indicated (***). Mean values and SEM are given. LDH = lactate dehydrogenase; TNF = tumor necrosis factor.

By using confocal live imaging in combination with Alexa 488 Shiga toxin subunit B (Stx-B), images show that Stx-B is located on the cell surface of healthy donor BOECs at 4 °C to prevent the internalization of Stx by inhibition of endocytosis (Figure 2A). At 37 °C there is internalization of the Stx-B into the cells. Images also show small parts of the glycocalyx inside the cell, which fits with the process of internalization of the toxin by endocytosis (Figure 2A). No cytotoxicity was seen in our set-up as only the B-unit of the holotoxin was used [18]. The binding of Stx-B on the cell surface was confirmed by flow cytometry analysis. In the past, it has been shown that endothelial cells become more sensitive for Stx when pre-incubated with inflammatory factors such as tumor necrosis factor alpha (TNF-α) and interleukin 1 (IL-1) due to upregulation of the Gb3 receptor [9,18]. Pre-incubation with TNF-α resulted in a three-fold increase in Stx-B binding (Figure 2B). A concentration range between 2.5 and 20.0 ng/mL for 24 h was used. No increased levels of Stx-B binding were found with higher concentrations of TNF-α (Figure 2B), likely explained by obtainment of full saturation of the receptor on the cell surface. To determine if this effect was TNF-α specific, BOECs were incubated with 1 ng/mL of IL-1α or 1 ng/mL of interleukin 6 (IL-6) for 24 h. Both TNF-α and IL-1α increased Stx-B cell surface binding, however IL-6 did not cause an effect (Figure 2C). Next, the cytotoxic effect of Stx2a on healthy donor BOECs was studied with the use of a lactate dehydrogenase (LDH) release assay. LDH is an intracellular enzyme that gets released when the cell membrane is compromised, and cell damage occurs. Results showed that Stx2a is cytotoxic for BOECs in a concentration-dependent manner over a time period of 48 h (Figure 3A). Vero cells were used as a control cell line due to their well-known sensitivity towards Stx [19,20]. Interestingly, it is known that some endothelial cells in vitro need pre-incubation with inflammatory factors such as TNF-α and IL-1 [9,18] to become sensitive for Stx, however this is not the case for the obtained BOECs (Figure 3A). To study if BOECs become even more sensitive after pre-incubation with inflammatory factors, BOECs were pre-incubated with 10 ng/mL TNF-α for 24 h and incubated with Stx2a for 24 h. This resulted in 20–30% additional LDH-release (Figure 3B), confirming that BOECs become more sensitive for Stx2a after pre-incubation with TNF-α. This is most likely explained by upregulation of enzymes involved in the Gb3 metabolic pathway, as shown by others [21].

### 2.3. Functional Consequences of Shiga Toxin on Healthy Donor BOECs

**Figure 4:**
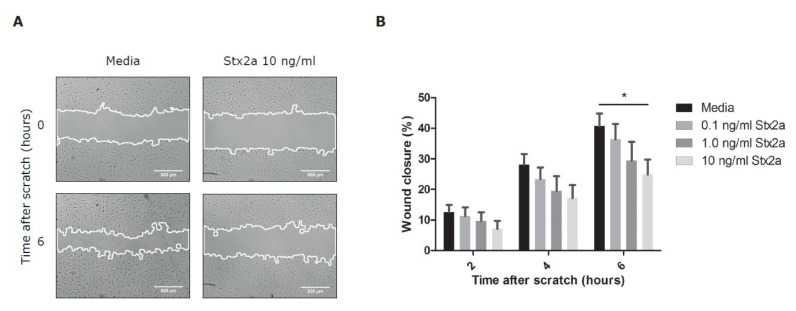
The effect of Stx2a on endothelial wound closure. Healthy donor BOECs were incubated with different concentrations of Stx2a for 24 h. A manual scratch wound was created, and images were taken after 0, 2, 4, and 6 h. (**A**) Representative images of a manual scratch wound in media and Stx2a conditions. (**B**) Bar graphs show the percentage of wound closure after incubation with media or different concentrations of Stx2a for 24 h (N = 9). Stx2a delayed endothelial wound closure in a concentration-dependent manner. Mean values and SEM are given. *p*-Value of < 0.05 (*) by t-test is indicated.

**Figure 5:**
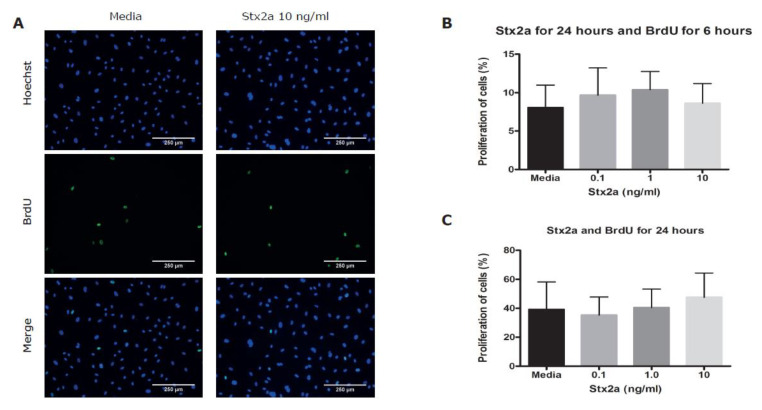
The effect of Stx2a on the proliferation of BOECs. (**A**) Healthy control BOECs were incubated with media or 10 ng/mL of Stx2a for 24 h in combination with BrdU to measure cell proliferation. Proliferating cells incorporate BrdU (green) in the nucleus (blue) (N = 4). (**B**) Bar graphs show analyzed results of images shown in Figure 5A. (**C**) Bar graphs show analyzed results of proliferation of BOECs after incubation with Stx2a and BrdU for 24 h without the creation of a scratch wound (N = 4). Stx2a did not significantly affect the proliferation of BOECs *(p* < 0.05). Mean values and SEM are given. BrdU = bromodeoxyuridine.

To study functional consequences of Stx2a on BOECs, a manual scratch wound assay was done. This assay is commonly used to investigate basic cell migration and cell repair mechanisms [22]. Stx2a concentrations in the 50% mortality range were chosen and selected from the results shown in Figure 3. Incubation with Stx2a delayed endothelial wound closure in a dose-dependent manner with a significant difference measured when 10 ng/mL of Stx2a was used, however the toxin did not fully inhibit wound closure (Figure 4). After 2 h, a difference in wound closure between control and Stx conditions was already detected although this was not significant. This difference likely cannot be explained by protein synthesis inhibition as this effect would have been expected after 4–6 h only and is strengthened by the fact that the area of the wound between control and Stx conditions was equally closed when absolute differences were calculated between the timepoints 2 and 6 h ((% wound closure at 6 h/% wound closure at 2 h) * 100 = 274% wound closure in media conditions versus 300% wound closure for 10 ng/mL of Stx2a). To study if a delayed wound closure could be explained by decreased endothelial cell proliferation, a bromodeoxyuridine (BrdU) incorporation assay was used. BrdU incorporates and replaces thymidine into newly synthesized DNA of proliferating cells [23]. During the scratch wound assay, BrdU was added to measure cell proliferation over a time course of 6 h. No significant differences in cell proliferation were found between BOECs incubated with or without Stx2a (Figure 5A,B). Cell proliferation was also investigated over a time period of 24 h without the creation of a manual scratch wound. Once again, incubation with Stx2a did not cause a significant difference on the proliferation of BOECs, which fits with the observed wound closure time curve (Figure 5C).

### 2.4. Effect of Stx on BOECs Originating from Controls and STEC-HUS Patients

**Figure 6:**
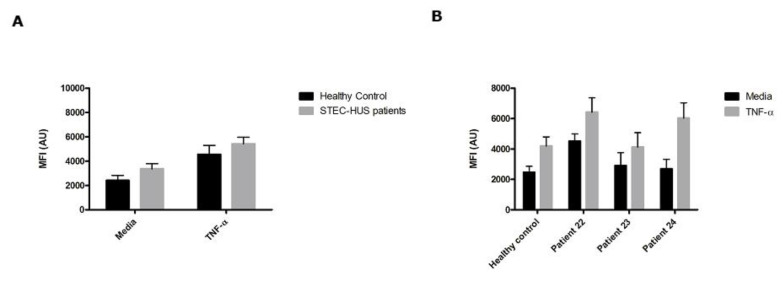
Binding of Shiga toxin subunit B on BOECs derived from STEC-HUS patients. (**A**) BOECs derived from 1 healthy control and 3 STEC-HUS patients were pre-incubated with or without 10 ng/mL TNF-α for 24 h (N = 4). The binding of Stx-B was measured with Alexa 488 labeled Stx-B in combination with flow cytometry. (**B**) Results as shown in (A) expressed as individual results. No significant differences in binding of Stx-B on the cell surface were found between BOECs from healthy donors and BOECs derived from patients with STEC-HUS *(p* < 0.05). Mean values and SEM are given. MFI = mean fluorescence intensity.

**Figure 7:**
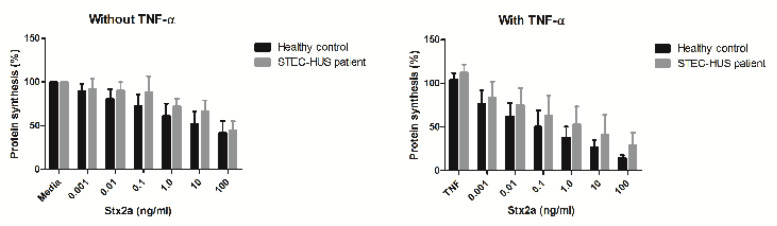
The effect of Stx2a on protein synthesis of BOECs derived from STEC-HUS patients. Healthy donor BOECs and BOECs from STEC-HUS patients were pre-incubated with or without 10 ng/mL of TNF-α and incubated with increasing concentrations of Stx2a for 24 h. Protein synthesis was measured with a ^3^H-leucine protein synthesis assay. Stx2a inhibits protein synthesis in a concentration-dependent manner and pre-incubation with TNF-α increased this effect. No difference was found between BOECs from healthy donors and BOECs from STEC-HUS patients. Mean values and SEM are given.

To test the hypothesis if BOECs derived from STEC-HUS patients bind more Stx, we used Alexa 488 Stx-B in combination with flow cytometry. STEC-HUS was clinically diagnosed and BOECs were isolated from three patients (Table 1). No significant differences in Stx-B binding levels were found between the different donors (Figure 6). Pre-incubation with 10 ng/mL of TNF-α re-confirmed increased Stx-B binding. As Stx-B binding was similar between the control and STEC-HUS BOECs, we investigated if this also resulted in similar cytotoxicity levels for Stx2a tested with a ^3^H-leucine protein synthesis assay. BOECs from three healthy donors and two patients with STEC-HUS were investigated. First, the results show protein synthesis inhibition by Stx2a in a concentration-dependent manner comparable with the results of the LDH assay. Second, this effect was enhanced by pre-treatment with 10 ng/mL of TNF-α. Third, there was no difference on the inhibition of protein synthesis between BOECs from healthy donors and BOECs from patients with STEC-HUS in the presence of various concentrations of Stx2a without and with TNF-α (Figure 7). This is in line with the results of the Stx-B binding assay (Figure 6).

## 3. Discussion

In this study, the interaction of Stx2a with healthy donor BOECs was investigated. BOECs provide the unprecedented opportunity to study the characteristic of the endothelium of individuals. Around 15% of children infected with Stx develop HUS, however, the reason why certain children develop this disease and others do not is still unknown. We showed that Stx is able to bind to the cell surface of BOECs and that Stx2a is cytotoxic for BOECs in a dose-dependent manner. Interestingly, BOECs are already highly susceptible for Stx2a, even without the pre-stimulation with pro-inflammatory cytokines. Nonetheless, pre-incubation with TNF-α or IL-1α did result in increased Stx binding with 20–30% more LDH release. Significantly delayed endothelial wound healing was found in a Stx2a-rich environment, although this was not caused by an effect on the proliferation rate of BOECs. We hypothesized that BOECs from STEC-HUS patients would be more susceptible for Stx and bind more Stx on the cell surface with higher cytotoxicity as a result than control BOECs. No significant differences were found between BOECs from healthy controls and BOECs from patients recovered from acute STEC-HUS in terms of Stx-B binding and inhibition of protein synthesis in static conditions.

In the past, expression of the Gb3 receptor and sensitivity for Stx on different endothelial cell lines and primary endothelial cells has been shown [9,10,18,24,25,26]. Confocal live imaging and flow cytometric analysis using Alexa 488 labeled Stx-B detects both Gb3 and Gb4 [27] and measurement of Stx-B binding to the cell surface is an indirect way to show the presence of both receptors. However, it is very likely that BOECs express in particular the Gb3 receptor as Stx is only capable of weakly binding to Gb4 [28]. It seems that BOECs are just as sensitive for Stx2a as Vero cells, a culture known to be highly sensitive for Stx. In contrast, primary glomerular microvascular endothelial cells (GMVECs) and HUVECs both need pre-stimulation with inflammatory cytokines like TNF-α to become sensitive for Stx [9]. This is not the case for BOECs, although they did become more sensitive when TNF-α was used, probably caused by an upregulation of Gb3 on the cell surface.

Pre-incubation of BOECs with the interleukins TNF-α and IL-1α resulted in increased binding of Stx on the cell surface with more cytotoxicity and less protein synthesis. No effect in Stx-B binding was measured when IL-6 was used. The major function of IL-1 is the induction of pro-inflammatory proteins, while IL-6 is important for synthesis of acute phase proteins and leukocyte trafficking [29]. Different interleukins have different target cells. The endothelium is one of the principal targets of IL-1, while this is not the case for IL-6 [29,30,31]. The underlying pathways involved in Gb3 synthesis were not investigated, but others have shown that the upregulation of Gb3 expression after contact with inflammatory cytokines is a result of stimulation of enzymes involved in the Gb3 metabolic pathway [21,32].

Results of the scratch wound assay already showed delayed wound healing in the first hours after incubation with Stx2a. By surprise, incubation with Stx2a did not have a significant impact on the proliferation rate of BOECs. Literature on Stx and its effect on proliferation is scarce, though it has been shown that Stx inhibits proliferation of bovine lymphocytes [33] and human astrocytoma cells [34]. Another explanation for delayed wound healing could be an effect of Stx on cytokine, growth factor, and reactive oxygen species (ROS) levels. Different cytokines, growth factors, and ROS are factors that have been suggested to influence wound repair [35].

No significant differences in Stx-B binding levels and protein synthesis were found between BOECs isolated from control or the three recovered STEC-HUS patients, although individual binding levels varied considerably. Of note, the healthy control BOECs used in this study were derived from adult individuals as—based on ethical considerations—isolation of control BOECs from healthy children was not available and, thus, age-based differences in expression levels could not be excluded. However, in the past our laboratory studied the expression levels of the Gb3 receptor and sensitivity for Stx between glomerular endothelial cells derived from pediatric patients and adults (data unpublished). No differences were found, which fits with the results in this study. BOECs were isolated after 3–5 months of recovery from STEC-HUS. It might be interesting to isolate BOECs during different time points of disease to investigate if there are differences in terms of Stx-B binding over time.

In this study, BOECs and their interaction with Stx under static conditions was investigated. As damage to the endothelium is still the hallmark of the pathogenesis of STEC-HUS and BOECs reflect the genetic characteristics of an individual patient, it would be a unique and useful tool to study host endothelial cell behavior differences under dynamic conditions.

## 4. Materials and Methods

### 4.1. Ethics

This study was approved by the Research Ethics Board of The Hospital for Sick Children Toronto, ON, Canada (REB, Number 1000039544). Written informed consent was obtained with a signature from all patients or parents/legal guardians of controls and patients whose BOECs were used in this study. Healthy controls were defined as those who were phenotypically negative for thrombotic microangiopathies (TMA), not biologically related to a TMA patient or family member, with no chronical illness and no acute disease in the recent past. Blood of patients with STEC-HUS was collected after 3–5 months of recovery from disease (Table 1). This study was executed in keeping with the regulations of the Declaration of Helsinki.

### 4.2. Reagents

Shiga toxin subtype 2a (Stx2a) was kindly provided by Dorothea Orth-Höller [36] or ordered from Phoenix Lab (Tufts Medical Center, Boston, MA, USA). Alexa 488 labeled Shiga toxin subunit B (Stx-B) was kindly provided by C. Lingwood, The Hospital for Sick Children, Toronto, ON, Canada [27].

### 4.3. Cell Culture

BOECs from healthy controls and 3 patients with STEC-HUS in the recent past were isolated by a standard protocol [17]. Cells were stained for the specific endothelial cell markers VWF and VECAD/CD144 to control endothelial origin. Sub-confluent cells (80–90%) passages 5–12 were used for all experiments. Vero cells (African green monkey renal epithelial cells) were grown in Dulbecco’s modified Eagle’s medium (DMEM) (Wisent Bio products, St. Bruno, QC, Canada) with 10% fetal bovine serum (FBS) (Wisent Bioproducts, QC, Canada).

### 4.4. Immunofluorescence Staining and Imaging

BOECs were seeded on 18 mm coverslips in 12-well plates (Sarstedt, Numbrecht, Germany). Cells were fixed with 4% paraformaldehyde (PFA) (Electron Microscopy Sciences, Hatfield, PA, USA) in phosphate-buffered saline (PBS) (Wisent Bioproducts, St. Bruno, QC, Canada). Immunofluorescence staining and imaging were done according to the manufacturer’s protocol (Thermo Fisher Scientific, Waltham, MA, USA). Rabbit anti-VWF (DakoCytomation, Glostrup, Denmark) and goat anti-VECAD (Santa Cruz, Dallas, TX, USA) dilution 1:100 were used as primary antibodies. Corresponding species-specific Alexa Fluor 488- and Fluor 555-labeled antibodies (Thermo Fisher Scientific, Waltham, MA, USA) dilution 1:500 were used as secondary antibodies. Images were recorded by spinning disk confocal microscopy (Olympus IX81, Olympus corporation, Tokyo, Japan) under control of Volocity software (PerkinElmer, Groningen, The Netherlands).

### 4.5. Confocal Live Imaging

BOECs were seeded on 18 mm coverslips in 12-well plates. Stx-B in a concentration of 1 ug/mL was added and stored in 4 °C for 10 min. Alexa 647-labeled wheat germ agglutinin (WGA) (Thermo Fisher Scientific, Waltham, MA, USA) dilution 1:750 was added and stored at 4 °C for 5 min. Imaging was done with the use of a spinning disk confocal microscope (Axiovert 200M Carl Zeiss system, Quorum Technologies, East Sussex, UK) in combination with a back-thinned, cooled charge-coupled device camera (Hamamatsu Photonics, Shizuoka, Japan). In order to study internalization of Stx, warm media was added, and images were captured immediately afterwards.

### 4.6. Flow Cytometry

BOECs were seeded at a density of 300,000 cells/well in 6-well plates. Cells were incubated with or without 2.5–20 ng/mL of TNF-α (TNF-α, expressed in HEK 293 cells, HumanKine, CAS 94948-59), 1 ng/mL of interleukin-1α (Sigma-Aldrich, Oakville, ON, Canada) or 1 ng/mL of interleukin-6 (Sigma-Aldrich, Oakville, ON, Canada) for 24 h. The protocol used was adapted from a standard protocol (Abcam, Cambridge, UK). Alexa 488-labeled Stx-B at a concentration of 20 µg/mL was used as conjugated primary antibody.

### 4.7. Lactate Dehydrogenase (LDH) Cytotoxicity Assay

Vero cells and BOECs were seeded in a density of 80,000 cells per well, grown in a 96-well plate (Sarstedt, Numbrecht, Germany). Cells were pre-stimulated with or without 10 ng/mL of TNF-α for 24 h and incubated with increasing concentrations of Stx2a in a range of 0.001–100 ng/mL for 24 or 48 h. A Pierce LDH cytotoxicity assay kit (Pierce Biotechnology, Rockford, IL, USA) was used according to the manufacturer’s protocol.

### 4.8. Endothelial Wound Closure Assay

BOECs were seeded in a 24-well plate (Sarstedt, Numbrecht, Germany) at a density of 80,000–120,000 cells per well. Cells were incubated with Stx2a in a concentration of 0.1–10 ng/mL. A manual scratch wound was made with a 10 uL pipette tip and a previously published protocol was followed [37].

### 4.9. Bromodeoxyuridine (BrdU) Proliferation Assay

BOECs were seeded in a 12-well plate at a density of 70,000 cells per well. After 24 h, Stx2a in a concentration of 0.1–10 ng/mL together with 10 µM BrdU were added and stored for 24 h in a 37 °C incubator with 5% CO_2_. Next, the BrdU assay was performed according to the manufacturer’s protocol (Abcam, Cambridge, UK).

### 4.10. Protein Synthesis by Radiolabeled ^3^H-Leucine Incorporation Assay

BOECs were seeded in 24-well plates and incubated with TNF-α and Stx2a as described above. ^3^H-leucine (PerkinElmer, Boston, MA, USA) was added and stored for 24 h in a 37 °C incubator with 5% CO_2_. Then, 10% trichloroacetic acid (TCA) was added and incubated for 1 h at 4 °C. Next, 0.3 M NaOH was added and stored overnight in a 37 °C incubator. Last, 1.55 M HCl and Ultima Gold (Perkin Groningen, The Netherlands) were added and samples were ready for counting with a scintillation counter.

### 4.11. Statistical Analysis

Data were analyzed by Student’s t-test or ANCOVA. A *p*-value of 0.05 was set as statistically significant. All statistical analyses were performed using GraphPad Prism (GraphPad Software, La Jolla, CA, USA) or SPSS (IBM, Chicago, IL, USA). Data are expressed as mean +/− SEM. All experiments were done in three-fold (N = 3), unless stated differently.

## Figures and Tables

**Table 1:** Clinical and laboratory features of the STEC-HUS patients used in this study. Data of the 3 STEC-HUS patients used in this study were collected during the acute phase of disease. STEC-HUS = hemolytic uremic syndrome with an infection with the Shiga toxin (Stx) producing *Escherichia coli*; LDH = lactate dehydrogenase.

Characteristics	Patient 1	Patient 2	Patient 3
**Gender (M/F)**	M	F	M
**Age (years)**	8	8	8
***E. coli*** **serotype**	Non-O157	O157	O157
**Neutrophils** **(1.5–9.0 × 10^9^/L)**	6.0	10.6	10.5
**Leucocytes** **(4.0–10.0 × 10^9^/L)**	9.0	16.8	13.8
**Platelets** **(150–400 × 10^9^/L)**	78	92	30
**Hemoglobin in g/L** **(106–132 g/L)**	95	96	78
**LDH** **(134–225 U/L)**	5563	6392	8238
**Creatinine in umol/L** **(25–50 umol/L)**	204	341	220
**Need for dialysis**	No	Yes	No
